# Senescent Stroma-Derived Glutamine: A Driver of Aggressiveness in Prostate and Ovarian Cancer Cells

**DOI:** 10.3390/cells15090770

**Published:** 2026-04-24

**Authors:** Giulia Lori, Caterina Mancini, Caterina Paffetti, Dayana Desideri, Erica Pranzini, Alice Santi, Manuela Leri, Alessio Biagioni, Matteo Benelli, Pietro Spatafora, Fedele Maria Manicone, Flavia Sorbi, Angela Leo, Massimiliano Fambrini, Sergio Serni, Francesca Magherini, Maria Letizia Taddei

**Affiliations:** 1 Department of Experimental and Clinical Medicine, University of Florence, Viale Morgagni 50, 50134 Florence, Italy; giulia.lori@unifi.it (G.L.); caterina.mancini@unifi.it (C.M.); dayana.desideri@unifi.it (D.D.); alessio.biagioni@unifi.it (A.B.); angela91leo@gmail.com (A.L.); 2 Department of Experimental and Clinical Biomedical Sciences—“Mario Serio”, University of Florence, Viale Morgagni 50, 50134 Florence, Italy; caterina.paffetti@unifi.it (C.P.); erica.pranzini@unifi.it (E.P.); alice.santi@unifi.it (A.S.); manuela.leri@unifi.it (M.L.); matteo.benelli@unifi.it (M.B.); flavia.sorbi@unifi.it (F.S.); massimiliano.fambrini@unifi.it (M.F.); 3 Department of Minimally Invasive and Robotic Urologic Surgery and Kidney Transplantation, Careggi Hospital University of Florence, 50134 Florence, Italy; pietro.spatafora@hotmail.it (P.S.); sergio.serni@unifi.it (S.S.); 4 Careggi University Hospital, University of Florence, 50134 Florence, Italy; fedelemaria.manicone@unifi.it

**Keywords:** therapy-induced senescence, fibroblasts, glutamine metabolism, cancer cell invasion, NRF2/ETS1 signaling

## Abstract

**Highlights:**

**What are the main findings?**

- Therapy-induced senescent fibroblasts secrete high levels of glutamine that enhance prostate and ovarian cancer cell invasion
- Stromal-derived glutamine promotes invasion and stem-like features in cancer cells via SLC1A5 upregulation and activation of a redox-dependent NRF2/ETS1 signaling axis.

**What are the implications of the main findings?**

- Senescent stroma represents a previously unappreciated metabolic source of extracellular glutamine fueling post-therapy tumor aggressiveness.
- Targeting glutamine metabolism may counteract therapy-induced pro-tumorigenic microenvironmental effects and reduce relapse risk.

**Abstract:**

Cancer progression is influenced by the dynamic interplay between tumor cells and the surrounding stromal microenvironment. Therapy-induced senescence (TIS) of stromal fibroblasts represents a common outcome of anticancer treatments, contributing to tumor progression through the senescence-associated secretory phenotype (SASP). While SASP cytokines promote cancer malignancy, the contribution of secreted metabolites from senescent cells remains poorly understood. Here, we investigate the role of senescent stromal metabolism in regulating prostate and ovarian cancer cell invasion. Conditioned media (CM) from TIS-induced human prostate (HPFs) and ovarian fibroblasts (HOFs) promote enhanced invasion of cancer cells. Invasion is partially preserved after exposure to boiled CM, suggesting a role for heat-stable metabolic factors. Metabolomic profiling of senescent fibroblasts-derived CM reveals a significant increase in Glutamine (Gln) levels, identifying senescent stromal fibroblasts as a previously unrecognized source of extracellular Gln in the tumor microenvironment (TME). Exposure of cancer cells to senescent CM increases Gln uptake, together with upregulation of the transporter SLC1A5 and increased intracellular Gln. This metabolic adaptation is associated with increased malignant phenotype including epithelial-to-mesenchymal transition (EMT) and stemness features. Extracellular Gln depletion, pharmacological inhibition of glutaminase-1 (GLS1) in cancer cells, or Gln synthetase (GS) silencing in fibroblasts markedly impair senescent fibroblasts CM-induced invasion, EMT markers expression, and stemness features in cancer cells. Stromal-derived Gln is associated with increased cancer cell invasion through activation of a redox-dependent NRF2/ETS1 signaling axis. Analysis of patient-derived transcriptomic datasets further suggests chemotherapy-associated upregulation of Gln metabolism and *ETS1* expression. These findings identify senescent stromal-derived Gln as a key metabolic driver of prostate and ovarian cancer aggressiveness and reveal a TIS-associated metabolic vulnerability that could be explored in future preclinical studies.

## 1. Introduction

Cancer progression is supported by the crosstalk between tumor cells and the surrounding stroma. In this context, it is known that senescent stromal cells contribute to the development of a pro-inflammatory milieu and the acquisition of aggressive traits by cancer cells [1]. In addition to classic senescence inducers, such as telomere shortening, oncogene activation, DNA damage, epigenetic changes, or oxidative stress [2], it has been shown that anticancer treatments can also induce cellular senescence (known as therapy-induced senescence, TIS) in both tumor and non-cancerous cells, thus contributing to many detrimental side effects of therapies [3]. In this context, the senescence-associated secretory phenotype (SASP), which comprises a broad spectrum of cytokines, chemokines, growth factors, proteases, and metabolites, profoundly influences the tumor microenvironment (TME) by modulating inflammation, remodeling the extracellular matrix, and altering the behavior of neighboring cells [4]. While initially senescence acts as a tumor-suppressive mechanism by halting the proliferation of damaged cells, the SASP can paradoxically promote tumor progression at later stages of the disease. In particular, factors released by senescent fibroblasts promote cancer cell proliferation, angiogenesis, immune evasion, enhance phenotypic plasticity, by fostering traits such as epithelial-to-mesenchymal transition (EMT) and stemness, which are associated with metastasis and therapy resistance [5,6].

Previously, we investigated the effects of chemotherapy on the stromal compartment of prostate and ovarian cancers, and demonstrated that anticancer chemotherapeutics, regardless of their specific mechanism of action, induce a senescent phenotype in patient-derived stromal prostate and ovarian fibroblasts thereby promoting the invasive potential of tumor cells through the SASP [7]. Given that senescent cells undergo extensive metabolic reprogramming to support survival and increased secretion of SASP factors [8], here we focused on a possible involvement of SASP-associated metabolic components in regulating tumor progression, which to date remains poorly explored. Beyond soluble factors and cytokines, extracellular metabolites released by the tumor stroma are increasingly recognized as key regulators of cancer progression, promoting proliferation, immune escape, and metastases [9,10]. In this context, we found that the amino acid Glutamine (Gln) is enriched in the conditioned media (CM) of TIS stromal senescent cells. Given the established role of Gln in fueling cancer metabolism and supporting anabolic processes [11], we hypothesized that stromal-derived Gln might contribute to the acquisition of aggressive traits in cancer cells. Accordingly, we demonstrated that the utilization of senescent-stromal-derived Gln by ovarian and prostate cancer cells is associated with increased metastatic potential and stem-like traits. Overall, our findings uncover a previously uncharacterized role of the metabolic component of the SASP, specifically Gln, in driving cancer cell invasion and stemness. These results identify TIS-derived Gln as a critical factor in tumor progression and highlight metabolic crosstalk between tumor and stroma as a potential therapeutic vulnerability.

## 2. Materials and Methods

### 2.1. Cell Lines

Human prostate (PC3) and ovarian (SKOV3) cancer cell lines were obtained from ATCC (PC3: CVCL_E2RM; SKOV3: CVCL_0532). Human Prostate Fibroblasts (HPFs) were obtained from tissue samples isolated during surgery from patients (average age 70) who underwent surgical treatment for lower urinary tract symptoms caused by benign prostatic hyperplasia (BPH). Human Ovarian Fibroblasts (HOFs) were extracted from healthy peritoneal tissue samples collected during cytoreductive surgery performed on patients (average age 66) with advanced ovarian cancer. Surgical explants were obtained in accordance with the Ethics Committee of the Azienda Ospedaliera Universitaria Careggi (Florence, Italy), for prostate tissues: num 16583_bio; for ovarian tissues: num 14780.

All cells were cultured in Dulbecco’s Modified Eagle Medium (DMEM) high glucose (4.5 g/L) (Euroclone, Pero, Italy), supplemented with 10% Fetal Bovine Serum (Euroclone), 2 mM L-Gln (Euroclone), 1% penicillin, and streptomycin (Euroclone). Cells were routinely grown at 37 °C in humidified atmosphere with 5% CO_2_.

To obtain HPFs and HOFs, a fragment of surgical explant was transferred into a Petri dish and cut into small pieces about 0.2 cm. Then, tissue fragments were transferred into a new cell culture dish and placed in a central stripe under the pressure of a sterilized slide. DMEM high glucose supplemented with 20% FBS, 2 mM L-Gln, 2% Penicillin/Streptomycin, 100 μg/mL Kanamycin (Merck Sigma, Darmstadt, Germany), and 2.5 μg/mL Amphotericin B (Euroclone) was added. After 20 days fibroblasts that had formed a monolayer were detached by trypsinization and routinely cultured.

### 2.2. Cell Treatments and Preparation of CM

To induce senescence, HPFs and HOFs were exposed to either 5 nM Docetaxel (MedChemExpress, Monmouth Junction, NJ, USA, HY-B0011) in dimethyl sulfoxide (DMSO) or 20 μM Cisplatin (Merck Sigma #5663-27-1) in H_2_O, respectively for 24 h, while DMSO or H_2_O was added in control samples. Then, the culture medium was changed, and cells were maintained in a complete medium for additional 6 days before experiments were conducted, unless noted otherwise. To collect CM, senescent and control cells obtained as previously described, were incubated for 24 h in starvation medium: DMEM high glucose (4.5 g/L) (Euroclone), 1% penicillin, and streptomycin (Euroclone). CM was collected, clarified by centrifugation for 10 min at 1000 rpm and used freshly or stored at −80 °C until use. Prostate and ovarian cancer cells were then incubated for 72 h with CM from HPFs or HOFs, respectively. To treat cancer cells, CM were normalized on HPFs or HOFs cell number. For boiled CM preparation, CM were collected from HPFs or HOFs, then boiled for 20 min and clarified by centrifugation for 10 min at 1000 rpm.

### 2.3. Senescence-Associated β-Galactosidase Staining

Fibroblasts were first fixed with 3% paraformaldehyde in PBS for 5 min followed by three washes in PBS. Subsequently, cells were incubated with Senescence-Associated β-Galactosidase (SA-β-Gal) staining solution containing 5 mM potassium ferrocyanide, 5 nM potassium ferricyanide, 150 mM NaCl, 2 mM MgCl_2_, 40 mM citric acid monohydrate, 1 mg/mL 5-bromo-4-chloro-3-indolyl β-D-galactopyranoside (Merck Sigma #B4252), adjusted to pH 6.0. The staining was carried out for 12 to 18 h at 37 °C in a non-humidified incubator under atmospheric CO_2_ conditions. Images were acquired from five randomly chosen fields and cells positive to (SA-β-Gal) staining were detected by the presence of an insoluble blue intracellular precipitate. Total and positive cells were counted using ImageJ imaging system (version 1.54s). Data show the ratio between positive SA-β-Gal cells and total cells per field.

### 2.4. Western Blotting

Cells were lysed at 4 °C with RIPA buffer (Thermo Fisher Scientific, Waltham, MA, USA, #89900) and supplemented with protease and phosphatase cocktail inhibitors (Merck Sigma). Following 20 min of lysis, cellular extracts were centrifuged for 10 min at 14,000 rpm and protein concentration quantified using Bicinchoninic Acid (BCA) assay (Euroclone). Equal amounts of total protein (20–30 μg) were separated by SDS-PAGE gels (BioRad, Hercules, CA, USA ) and transferred to PVDF membranes (BioRad). Membranes were blocked for 1 h at room temperature (RT) in 5% non-fat dry milk (Santa Cruz Biotechnology, Paso Robles, TX, USA) in PBS-Tween 0.1% and then incubated overnight at 4 °C with primary antibody against p21 (Santa Cruz Biotechnology, sc-271610), p16 (Santa Cruz Biotechnology, sc-56330), Gln synthetase (GS) (Cell Signaling, Danvers, MA, USA, D203F), NRF2 (Santa Cruz Biotechnology, sc-365949), ETS1 (Cell Signaling, B808A), HSP90 (Santa Cruz Biotechnology, sc-69703), β-Actin (Santa Cruz Biotechnology, sc-47778), Vinculin (Merck Sigma V9264). After washing, membranes were incubated for 1 h at RT with antirabbit horseradish peroxidase-conjugated (Santa Cruz Biotechnology #2357) or antimouse horseradish peroxidase-conjugated (Santa Cruz Biotechnology #516102). Proteins were visualized using Clarity Western ECL Substrate (BioRad) and images acquired using Amersham Imager 600 (GE Healthcare, Chicago, IL, USA). HSP90 or β-Actin was used as loading control. All western blot images are representative of at least three independent experiments. The densitometric analysis of all western blots reported in the Results (three independent experiments) is presented in Supplementary Figure S1.

### 2.5. Invasion Assay

Prostate cancer cells (8 × 10^4^) and ovarian cancer cells (10 × 10^4^) were seeded in 200 μL of starvation medium in the upper chamber of 8 μm pore Transwell (Greiner Bio-One, Kremsmünster, Austria) coated with 50 μg/cm^2^ Matrigel (Corning, New York, NY, USA). The lower chamber was filled with a complete medium containing 10% FBS as chemoattractant. After 16 h, cells that have invaded toward the lower surface of the filters were fixed and stained with Crystal Violet (Merck Sigma). Invasive capacity was quantified by counting the number of stained cells in 5 randomly selected microscopic fields, and results are presented as the mean number of invading cells per field.

### 2.6. Real-Time PCR

Total RNA was extracted from cells using RNeasy Plus Mini Kit (Qiagen, Hilden, Germany, #74134) according to the manufacturer’s instruction and quantified with NanoDrop Microvolume Spectrophotometer (NanoDrop Technologies LLC, Wilmington, DE, USA) and Fluorometer (Thermo Fisher Scientific). cDNA synthesis was obtained by incubating 1 μg of total RNA with High-Capacity cDNA Reverse Transcription Kit (Thermo Fisher Scientific) according to the manufacturer’s instructions. mRNA expression levels were quantified by Real-Time PCR using Luna Universal qPCR Master Mix (New England Biolabs, Ipswich, MA, USA).

The nucleotide sequences of the specific primers (Thermo Fisher Scientific) used were: EpCAM-FW 5′-TGTGGTGATAGCAGTTGTTGC-3′, EpCAM-REV 5′-CTATGCATCTCACCCATCTCC-3′; ECAD-FW 5′-AGGCCAAGCAGCAGTACATT-3′, ECAD-REV 5′-ATTCACATCCAGCACATCCA-3′; NCAD-FW 5′-CCTCCAGAGTTTACTGCCATGAC-3′, NCAD-REV 5′-GTAGGATCTCCGCCACTGATTC-3′; VIM-FW 5′-ACACCCTGCAATCTTTCAGACA-3′, VIM-REV 5′-GATTCCACTTTGCGTTCAAGGT-3′; ZEB1-FW 5′-AAGAAAGTGTTACAGATGCAGCTG-3′, ZEB1-REV 5′-CCCTGGTAACACTGTCTGGTC-3′; ZEB2-FW 5′-AGGGACAGA TCAGCACCAAA-3′; ZEB2-REV 5′-GTGCGAACTGTAGGAACCAG-3′; SNAIL-FW 5′-CCTCCCTGTCAGATGAGGAC-3′, SNAIL-REV 5′-CAAGGAATACCTCAGCCTGG-3′; SLUG-FW 5′-ACAGCGAACTGGACACACAT-3′, SLUG-REV 5′-GATGGGGCTGTATGCTCCT-3′; B2M-FW 5′-AGTATGCCTGCCGTGTGAAC-3′, B2M-REV 5′-GCGGCATCTTCAAACCTCCA-3′.

qRT-PCR was performed using CFX96 Real-Time PCR System (BioRad). Data were reported as relative quantity with respect to the reference samples using 2^−ΔΔCt^. Data were normalized on β2-microglobulin.

### 2.7. Total ROS Quantification

Intracellular reactive oxygen species (ROS) levels were assessed using the fluorescent probe 2′,7′–dichlorofluorescin diacetate (DCFDA, Merck Sigma, #287810). Cells were detached, pelleted by centrifugation, and resuspended in a staining solution containing 5 µM DCFDA. Samples were incubated for 30 min at 37 °C protected from light. Following incubation, cells were washed to remove excess dye and resuspended in PBS. Flow cytometry analysis was performed using a BD FACS Canto II cytometer (BD Biosciences, Franklin Lakes, NJ, USA). The viable cell population was gated based on morphological parameters using Forward Scatter (FSC) and Side Scatter (SSC). Fluorescence was detected in the FITC channel (488 nm excitation laser; 530/30 nm bandpass filter). Background fluorescence was determined using unstained controls to define the threshold for positivity. Data were acquired and analyzed as both the percentage of positive cells and the Mean Fluorescence Intensity (MFI).

### 2.8. Prostate and Ovarian Sphere Formation

Cells were grown in anchorage-independent conditions in poly-hydroxyethylmethacrylate (poly-HEMA)-coated dishes (Merck Sigma, #P3932) with selective serum-free DMEM/F12 medium supplemented with 50× B27 (Gibco, Waltham, MA, USA), 20 ng/mL bFGF (Bio-Techne, Minneapolis, MN, USA), 20 ng/mL EGF (Relia Tech, Wolfenbüttel, Germany). For PC3, sphere medium was also supplemented with N2 Supplement 100× (Gibco, #17502-048). Cancer cells were incubated for 72 h with CM from HPFs and HOFs, then 700 cells/well (PC3) or 1000 cells/well (SKOV3) were seeded in a 96-well plate precoated with poly-HEMA. After 7 days, photos were taken to determine the volume of spheres. Data were reported as the average volume of formed spheres/field, in at least 5 randomly chosen fields. Spheroids volume was calculated measuring length (L) and height (H) with ImageJ using the following formula: V = (L^2^ × H)/2, as previously reported [12].

### 2.9. Cell Transfection

Control siRNA (SIC001) and GS siRNA (#HS02_00307974) were purchased from Merck Sigma. Senescent cells were transfected with 45 nM siRNAs at approximately 70% confluence with RNAiMAX (Thermo Fisher Scientific) according to manufacturer’s instructions. GS expression was assessed by western blotting 48 h after transfection.

### 2.10. Cell Viability

Cells (6 × 10^3^) were seeded in 100 μL of complete culture medium in 96-well plates and allowed to adhere for 24 h prior to treatment. After the treatment, cells were washed with PBS and 5 mmol/L MTT (3-(4,5-Dimethylthiazol 2-yl)-2,5-diphenyltetrazolium bromide, Merck Sigma) was added for 1 h at 37 °C. The resulting formazan crystals were dissolved in 200 µL of DMSO, and absorbance was measured at 595 nm using a spectrophotometer MULTISKAN FC (Thermo Fisher Scientific).

To evaluate the effect of Gln deprivation, 15 × 10^4^ cells were seeded in 35 mm dishes and incubated with or without 2 mM Gln (Euroclone) for 72 h. Then cells were detached and incubated with LIVE/DEAD Violet Kit (Thermo Fisher Scientific, #L34964A) for 30 min at RT according to the manufacturer’s protocol. The dye was reconstituted in 50 µL of DMSO and diluted 1:500 in PBS to create a working solution. Following a single wash with PBS, samples were analyzed via flow cytometry using a BD FACSCanto II. The violet dye (excitation 405 nm; emission 450 nm) was detected using a BV421/DAPI filter, allowing for clear discrimination between unstained live cells and stained dead cells.

### 2.11. Determination of GSH/GSSG

The intracellular reduced (GSH) and oxidized (GSSG) glutathione levels were measured using the GSH/GSSG-Glo™ Assay (Promega, Fitchburg, WI, USA V6611), according to the manufacturer’s instructions. Briefly, cells were lysed and total glutathione (GSH + GSSG) was quantified through the conversion of a GSH probe, Luciferin-NT, to luciferin by a glutathione S-transferase enzyme. To selectively measure GSSG, GSH was first derivatized using the provided masking reagent, allowing specific detection of GSSG. Luminescence was measured using a Sinergy H1 plate reader (BioTek Winooski, Winooski, VT, USA), and GSH concentrations were calculated from standard curves. Values were normalized to protein content.

### 2.12. Determination of Gln and Ammonium Levels

Gln and ammonium levels were determined using the L-Glutamine/Ammonia Assay Kit (Rapid) (Megazyme, Bray, Ireland, K-GLNAM), according to the manufacturer’s instructions. Briefly, the Gln levels were measured in culture media through an enzymatic conversion of Gln to glutamate (Glu) and ammonium by glutaminase (GLS). Ammonium was subsequently quantified through a glutamate dehydrogenase–coupled reaction by monitoring NADPH consumption as a decrease in absorbance at 340 nm with an uv-1800 shimadzu (Shimadzu Corporation, Kyoto, Japan) spectrophotometer.

### 2.13. Gas Chromatography–Mass Spectrometry (GC–MS) Analysis

For total metabolites quantification, media from HPFs and HOFs were collected, and 50 μL were mixed with 50 μL of cold 80% methanol in HPLC-grade water containing internal standards (1 µg/mL norvaline and 1.25 µg/mL glutaric acid).

For isotope tracing experiments, fibroblasts were cultured in MEM (Gibco) supplemented with 1% MEM vitamins (Merck Sigma), 1% penicillin-streptomycin (Euroclone), 0.4 mM glycine (Merck Sigma), 0.4 mM serine (Merck Sigma), and 17 mM [U − ^13^C] glucose (Cambridge Isotope Laboratories, Inc. Andover, MA, USA). After 24 h, CM was collected. One aliquot of CM was used for metabolites extraction and GC–MS analysis, as described above. Metabolites were also extracted from fibroblast cell lysates.

The remaining labeled CM was used to treat PC3 cells for 24 h, after which metabolites were extracted from PC3 cells for downstream analysis. To extract intracellular metabolites, fibroblasts and PC3 cells were washed twice with 0.9% NaCl at 4 °C and scraped in 400 µL of cold 80% methanol in HPLC water containing 1 µg/mL norvaline and 1.25 µg/mL glutaric acid as internal standards. Samples were sonicated on ice for 5 s for three times with a 5 s interval at 70% amplitude, centrifugated at 14,000 rpm, 4 °C for 10 min, and supernatants were collected. Samples were dried by using a vacuum concentrator (Labconco, Kansas City, MO, USA). Dried extracts were derivatized with 10 µL of 40 mg/mL methoxyamine hydrochloride (Merck #226904) in pyridine (Merck #270970) for 90 min at 37 °C. Then, 50 µL of N-(tert-butyldimethylsilyl)-N-methyl-trifluoroacetamide, with 1% tert-butyldimethylchlorosilane (Merck Sigma #375934) were added and samples were incubated for an additional 30 min at 60 °C. GC-MS runs were performed by using an Intuvo 9000 GC/5977B MS System (Agilent Technologies, Folsom, CA, USA) equipped with an HP-5MS capillary column (30 m × 0.25 mm × 0.25 µm). 1 µL of each sample was injected in splitless mode using an inlet liner temperature of 240 °C. GC runs were performed with helium as carrier gas at 1 mL/min. The GC oven temperature ramp was from 70 °C to 280 °C. The temperature of 70 °C was held for 2 min. Then, the first temperature ramp was from 70 °C to 140 °C at 3 °C/min. The second ramp was from 140 °C to 150 °C at 1 °C/min. The third temperature ramp was from 150 °C to 280 °C at 3 °C/min. Metabolites were detected using electron impact ionization at 70 eV using a SIM mode. The ion source and transfer line temperature were set to 230 °C and 290 °C, respectively.

Quantitative analysis was performed using MS Quantitative Analysis software (Agilent Technologies, version 10.2). Relative metabolite abundances were calculated by integrating the signal of selected ion for each metabolite and normalizing to the signal of the internal standards (norvaline or glutaric acid) and to protein content. For isotopic labeling experiments, all the measured values were corrected for ^13^C natural abundance by using IsoCorrectoR [13].

### 2.14. Confocal Immunofluorescence

Sub-confluent PC3 and SKOV3 cells were cultured on glass coverslips and exposed for 72 h to CM derived from HPFs or HOFs. Where indicated, the GLS1 inhibitor BPTES (Bis-2-(5-phenylacetamido-1,2,4-thiadiazol-2-yl) ethyl sulfide) was added at a final concentration of 1 µM during the final 16 h of incubation. At the end of the incubation period, nuclei were stained with DAPI (Sigma Aldrich, D9542) for 20 min at 37 °C. ETS1 protein was detected using a rabbit monoclonal anti-ETS1 (1:1000, Cell Signaling, #14069), followed by antirabbit Alexa Fluor 568-conjugated secondary antibodies (Thermo Fisher Scientific, A-11011, red channel). Cell fluorescence was imaged using a Leica TCS SP8 confocal scanning microscope (Leica, Mannheim, Germany; Durham, NC, USA; Danaher, Washington, DC, USA). Observations were made with a Leica HC PL Apo CS2 X63 oil immersion objective. Images reconstruction and signal fluorescence quantification was obtained using Image J Fiji software [14].

### 2.15. Statistical Analysis

Statistical analysis of the data was performed using unpaired, two-tailed Student *t*-test or one-way ANOVA followed by Tukey’s post hoc test for multiple comparisons, with GraphPad Prism version 8.0 (GraphPad Software). Data were expressed as the mean ± SEM. A *p*-value ≤ 0.05 was considered statistically significant. All the statistical analyses were carried out on three biological replicates. Statistical analysis of gene expression datasets from ovarian cancer patients was performed using a paired, two-tailed Wilcoxon signed-rank test.

## 3. Results

### 3.1. The Metabolic Component of CM Derived from Senescent Fibroblasts Supports Ovarian and Prostate Cancer Cell Invasion

**Figure 1:**
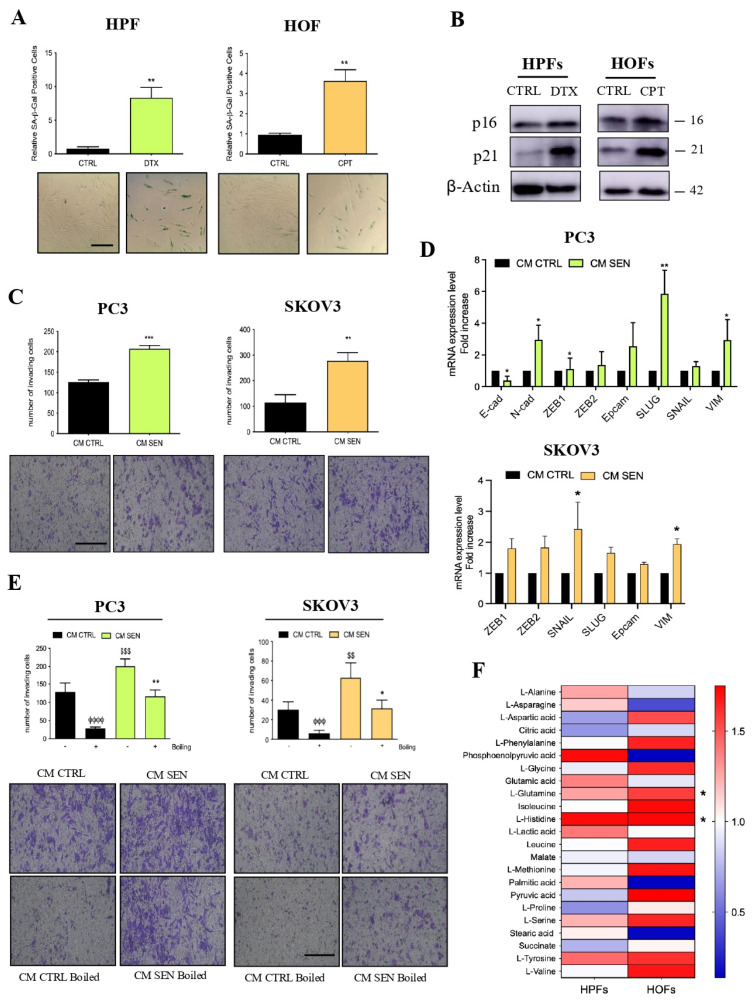
The metabolic component secreted by senescent HPFs and HOFs is involved in the acquisition of a pro-invasive phenotype in prostate and ovarian cancer cells. (**A**) Quantification of SA-β-Gal staining of human prostate fibroblasts (HPFs) and human ovarian fibroblasts (HOFs) treated for 24 h with 5 nM Docetaxel (DTX) and 20 μM cisplatin (CPT), respectively, and then cultured in drug-free medium for an additional 6 days. For each condition, images were taken from five randomly selected fields, and both the total number of cells and the number of blue (SA-β-Gal positive) cells were counted. Bar graphs represent the average ratio of positive cells to the total cell count. Representative images of the stained cells are shown below the bar graphs (magnification 20×, scale bar 100 μm). (**B**) Representative immunoblots of p16 and p21 protein levels in DTX-treated HPFs and CPT-treated HOFs. β-actin was used as loading control. (**C**) Invasion assay: PC3 and SKOV3 cells were incubated with CM CTRL and CM SEN conditioned media (CM) from senescent and non-senescent (CTRL) fibroblasts for 72 h and then seeded in Boyden chambers. Cells were allowed to invade for 16 h. Representative images of the filters are shown below the bar graphs (magnification 20×, scale bar 100 μm). (**D**) mRNA expression level of EMT key genes in PC3 and SKOV3 cells incubated with CM CTRL or CM SEN for 72 h. (**E**) Invasion assay: PC3 and SKOV3 cells were incubated for 72 h with boiled and non-boiled CM CTRL or CM SEN and then allowed to invade for 16 h. Representative images of the filters are shown below the bar graphs (magnification 20×, scale bar 100 μm). (**F**) GC–MS analysis of metabolites in CM-CTRL or CM-SEN. Data reported are normalized to CM CTRL. * symbol indicates metabolite levels significantly increased both in senescent CM from HPFs and HOFs. Data are means ± SEM of three independent experiments. Statistical significance was assessed by unpaired Student *t*-test (**A**,**C**,**D**,**F**) or one-way Anova followed by Tukey’s multiple comparisons (**E**). * *p* < 0.05; ** *p* < 0.01; *** *p* < 0.001; (^ϕϕϕ^ *p* < 0.001; ^ϕϕϕϕ^ *p* < 0.0001; ^$$^ *p* < 0.01; ^$$$^ *p* < 0.001). In panel (**E**), * indicates CM SEN boiled vs. SEN non-boiled; ^ϕ^ indicates CM CTRL boiled vs. CM CTRL non-boiled; ^$^ indicates CM SEN vs. CM CTRL.

As previously reported [7], chemotherapy treatment with Docetaxel (DTX) and Cisplatin (CPT) is able to induce TIS in human fibroblasts isolated from surgical explants of patients affected by benign prostatic hyperplasia (HPFs) or from healthy peritoneal tissues obtained during cytoreductive surgery for ovarian cancer (HOFs) [7]. Here, we extended the analysis to fibroblasts derived from newly established primary cultures obtained from eight ovarian and ten prostate cancer patients. All experiments were performed on fibroblasts derived from at least three different patients with prostatic hyperplasia or ovarian cancer. We first confirmed that chemotherapy treatment induces TIS in fibroblasts as shown by SA-β-Gal staining and expression of senescence markers (Figure 1A,B and Supplementary Figure S1). Moreover, CM from senescent fibroblasts sustain the acquisition of increased invasive abilities (Figure 1C) and increased expression of specific markers of EMT (Figure 1D) in PC3 and SKOV3 cancer cells.

To investigate the potential contribution of cytokine-independent components of SASP in conferring increased aggressiveness to cancer cells, we attempt to reduce the contribution of protein components by boiling senescent fibroblasts-derived CM. Interestingly, we observed that both PC3 and SKOV3 cells partially retain the ability to invade after the incubation with boiled CM (Figure 1E). While boiling is expected to markedly impair protein structure and function, we cannot exclude a residual contribution of protein components. Nevertheless, these observations are consistent with a potential role of non-protein factors, such as a secreted metabolite in the induction of this aggressive trait in cancer cells.

To further explore the contribution of stromal-derived metabolites to the pro-tumorigenic effects of senescent CM, we performed a metabolomic profiling of CM derived from senescent and control HPFs and HOFs by GC–MS. This analysis identified a subset of metabolites whose abundance was significantly altered in CM from senescent fibroblasts compared with their non-senescent counterparts (Figure 1F). Notably, some of these metabolites were commonly deregulated across both prostate and ovarian stromal models, indicating the existence of conserved metabolic alterations associated with stromal senescence. Interestingly, among these shared metabolites, Gln emerged as one of the most significantly enriched in senescent cell-derived CM from both cellular models (see Supplementary Figure S2 for metabolite abundance relative to cell number in the two different models). Given the well-established Gln addiction of multiple tumor types [15], we focused our subsequent analyses on Gln as a potential key metabolic mediator of the pro-invasive and pro-aggressive effects exerted by senescent cell-derived CM on prostate and ovarian cancer cells.

### 3.2. Availability of Senescent Stroma-Derived Gln Drives Invasive Abilities of PC3 and SKOV3 Cells

**Figure 2:**
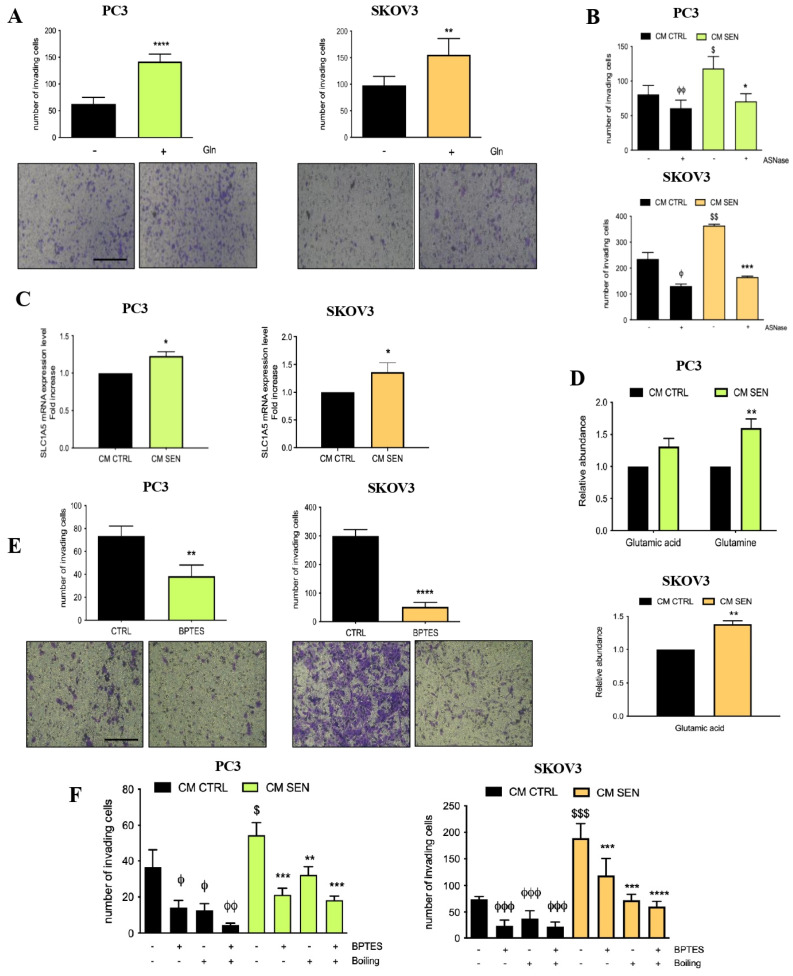
Gln availability drives invasive ability in PC3 and SKOV3 cells. (**A**) Invasion assay: PC3 and SKOV3 cells were incubated for 72 h with DMEM in presence or absence of 2 mM Glutamine (Gln) and then seeded in Boyden chambers. Representative images of the filters are shown below the bar graphs (magnification 20×, scale bar 100 μm). (**B**) Invasion assay: PC3 and SKOV3 were incubated with CM CTRL and CM SEN for 72 h and with ASNase 1 U/mL during the last 48 h. Then, cells were seeded in Boyden chambers; (**C**) mRNA expression level of Gln transporter SLC1A5 in PC3 and SKOV3 cells incubated with CM CTRL or CM SEN for 72 h. (**D**) PC3 and SKOV3 cells were incubated for 72 h with CM CTRL or CM SEN. Then, GC–MS analysis was performed to measure Glu and Gln intracellular content. (**E**) Invasion assay: PC3 and SKOV3 cells were incubated for 72 h with DMEM and then seeded in a Boyden chamber in presence or absence of BPTES 1 μM for 16 h. Representative images of the filters are shown below the bar graphs (magnification 20×, scale bar 100 μm). (**F**) Invasion assay: PC3 and SKOV3 cells were incubated for 72 h with boiled and non-boiled CM; BPTES 1 μM was added during the final 16 h of incubation. Data are means ± SEM of three independent experiments. Statistical significance was assessed by Student *t*-test (**A**,**C**–**E**) or one-way Anova followed by Tukey’s multiple comparisons (**B**,**F**). * *p* < 0.05; ** *p* < 0.01; *** *p* < 0.001; **** *p* < 0.001; ^ϕ^ *p* < 0.05; ^ϕϕ^ *p* < 0.01; ^ϕϕϕ^ *p* < 0.001; ^$^ *p* < 0.05; ^$$^ *p* < 0.01; ^$$$^ *p* < 0.001. In panel (**E**), * indicates CM SEN + ASNase vs. CM SEN; ^ϕ^ indicates CM CTRL + ASNase vs. CM CTRL; ^$^ indicates CM SEN vs. CM CTRL. In panel (**F**), * indicates treated CM SEN vs. CM SEN, ^ϕ^ indicates treated CM CTRL vs. CM CTRL, ^$^ indicates CM SEN vs. CM CTRL.

We first tested the effect of Gln supplementation on prostate and ovarian cancer cell invasion and we found that it markedly enhanced the invasive properties of PC3 and SKOV3 cells (Figure 2A) to a similar extent as observed following conditioning of cancer cells with CM from senescent cells (Figure 1C), suggesting that Gln alone can really drive the invasive abilities of cancer cells. As a complementary approach, we depleted Gln from senescent fibroblast-derived CM by exploiting the glutaminase activity of L-asparaginase (ASNase) (see Supplementary Figure S3A and [16]) and observed a marked reduction in cancer cell invasion following incubation with ASNase-treated senescent CM (Figure 2B). Notably, we showed that either lack of Gln or ASNase treatment do not affect cell viability as shown in Supplementary Figure S3B,C.

Thus, we hypothesized that senescent stromal derived-Gln could be exploited by cancer cells to fuel their aggressive behavior. To investigate whether Gln released by senescent fibroblasts contributes to cancer-cell-specific response, we incubated PC3 and SKOV3 cancer cells with senescent cell-derived CM. Subsequently, mRNA levels of the Gln transporter SLC1A5 were quantified by RT-PCR, and intracellular levels of Gln and Glu were measured by GC–MS. We observed that the treatment with CM derived from senescent fibroblasts resulted in increased expression of SLC1A5 (Figure 2C) and enhanced intracellular content of Gln and Glu (Figure 2D). Notably, even if in ovarian cancer cells intracellular Gln was not detectable, we coherently measured a significant increase in Glu abundance.

Moreover, to investigate the role of Gln in tumor cells, we evaluated the effect of incubating cells in the presence of BPTES, which is a specific and selective inhibitor of GLS1 and Gln metabolism [17]. Indeed, BPTES, preventing Gln utilization by cancer cells, reduced cell invasion (Figure 2E) without affecting cell viability (see Supplementary Figure S3D). Besides, to confirm the role of senescent stromal-derived Gln on the invasive abilities of prostate and ovarian cancer cells, we incubated PC3 and SKOV3 cells with boiled CM in the presence of the BPTES inhibitor. We found that the impairment of Gln utilization severely decreased the invasive abilities of both cancer cell lines (Figure 2F). Together, these data underline the powerful role of Gln utilization to drive the invasion of cancer cells.

**Figure 3:**
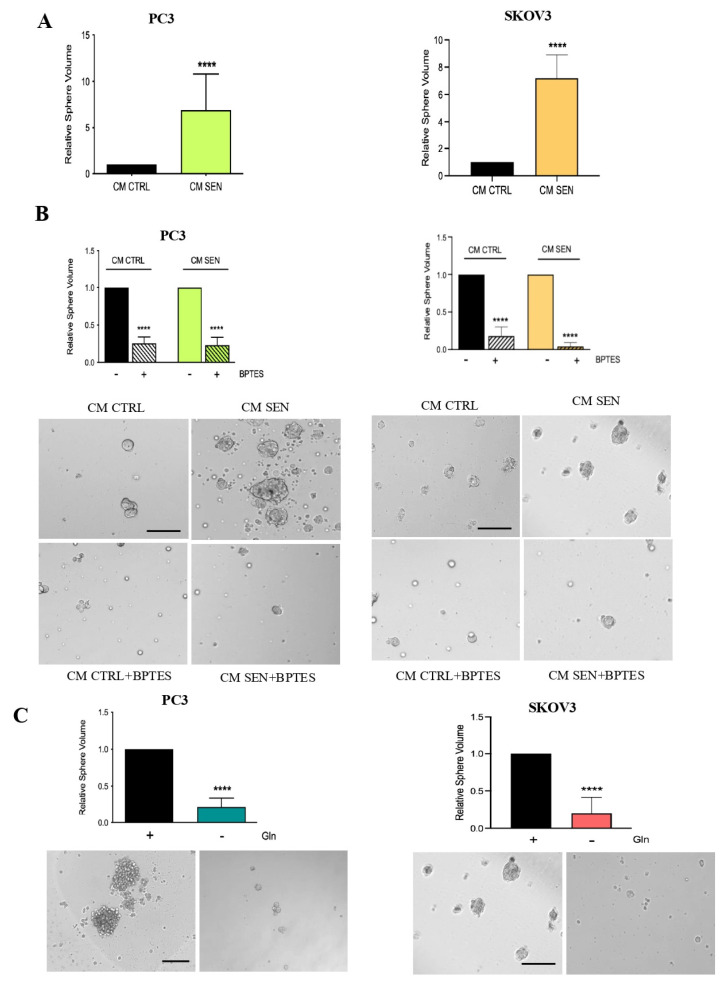
Gln confers aggressiveness to prostate and ovarian cancer cells. (**A**,**B**) PC3 and SKOV3 cells were conditioned for 72 h with CM from HPFs and HOFs; BPTES 1 μM was added during the final 16 h of incubation. Then, cells were detached and grown as spheroids for 7 days. Volumes of prostatic and ovarian spheroids were calculated as described in M and M. Representative images of spheroids are shown below the bar graphs (magnification 20×, scale bar 100 μm). (**C**) Prostate and ovarian cancer cells were incubated for 72 h with or without 2 mM Gln. Then, cells were detached and grown as spheroids for 7 days. Volumes of prostatic and ovarian spheroids were calculated as described in M and M. Representative images of spheroids are shown below the bar graphs (magnification 20×, scale bar 100 μm). Data are represented as mean ± SEM of three independent experiments. Statistical significance was assessed by *t*-test. **** *p* < 0.0001.

Furthermore, since invasion and the development of the EMT program are closely related to the acquisition of stemness features in cancer cells [18], we analyzed whether senescent stromal-derived Gln could induce a stem cell phenotype in tumor cells. Thus, we evaluated the ability of tumor cells to form prostate and ovarian spheres following incubation with senescent cell-derived CM. As shown in Figure 3A, CM from senescent fibroblasts significantly enhanced the volume of tumor spheres, indicating increased self-renewal capacity. Notably, this effect was markedly impaired by treatment with BPTES (Figure 3B) suggesting the central role of Gln utilization in the achievement of stemness features by cancer cells. Consistently, Gln depletion from the culture medium strongly impaired sphere formation (Figure 3C).

Collectively, these data strongly support that senescent stroma-derived Gln is capable to sustain the aggressiveness of prostate and ovarian cancer cells.

### 3.3. Senescent Stroma Upregulates Gln Synthetase, Sustaining Gln Metabolism

**Figure 4:**
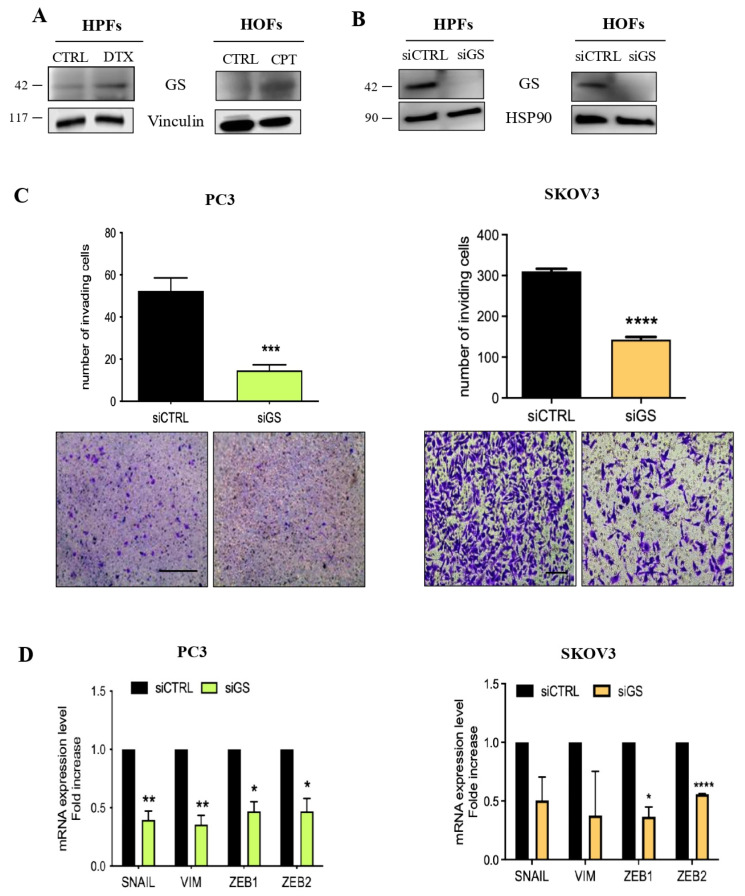
GS silencing in senescent fibroblasts affects EMT program in prostate and ovarian cancer cells. (**A**) Immunoblot of GS protein levels in senescent and CTRL fibroblasts. Vinculin was used as loading control. (**B**) GS protein levels in senescent HPFs and HOFs following 48 h gene silencing. HSP90 immunoblot was performed to ensure equal loading. (**C**) Invasion assay: PC3 and SKOV3 cells were incubated for 72 h with CM from GS silenced HPFs and HOFs and then let to invade for 16 h. Representative images of filters are shown below the bar graphs (magnification 20×, scale bar 100 μm). (**D**) mRNA expression level of EMT markers in PC3 and SKOV3 cells after 72 h incubation with CM from GS-silenced fibroblasts. Data are mean ± SEM of three independent experiments. Statistical significance was assessed by Student *t*-test. *t*-test * *p* < 0.05; ** *p* < 0.01; *** *p* < 0.001; **** *p* < 0.0001.

**Figure 5:**
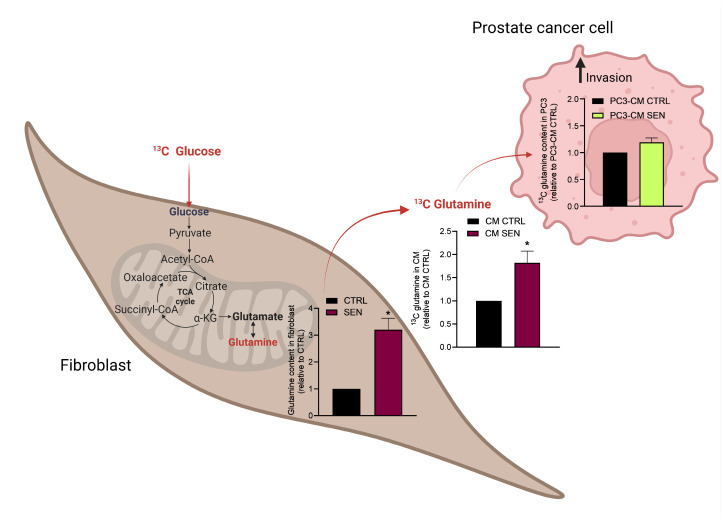
Senescent stroma-derived Gln is uploaded by PC3. CTR and senescent HPFs were incubated for 24 h with ^13^C glucose, then cell lysates and CM were collected for GC–MS analysis. PC3 cells were incubated with CM for further 24 h and the amount of ^13^C Gln inside cells was measured. Data are represented as mean ± SEM of three independent experiments. Statistical significance was assessed by Student *t*-test. * *p* < 0.05.

To gain insight into the molecular mechanisms responsible for the increased Gln production observed in senescent fibroblasts, we investigated whether senescent stroma could reprogram its metabolism to sustain Gln synthesis. Indeed, we observed an increase of Gln synthetase (GS) levels in prostate and ovarian senescent fibroblasts (Figure 4A and Supplementary Figure S1). To better understand the role of senescent stroma-derived Gln in cancer cells, we inhibited GS expression by small interfering RNA (siRNA) (Figure 4B and Supplementary Figure S1). CM collected from GS silenced-senescent fibroblasts showed a decreased ability to induce prostate and ovarian cell invasion (Figure 4C) as well as a decreased expression levels of mRNA for EMT markers in cancer cells (Figure 4D). These data provide evidence that senescent stroma really reprograms its metabolism and sustains cancer cell invasion. Moreover, to clarify whether cancer cells upload and exploit stroma-derived Gln, we traced the fate of Gln originating from the stroma. To do this, we treated HPFs with uniformly labeled ^13^C-glucose and then we followed the destiny of ^13^C-glucose-derived Gln. In particular, we quantified the amount of labeled Gln that was synthetized by fibroblasts and released into the CM; then, PC3 cells were incubated for 24 h with CM and the amount of labeled Gln was analyzed by GC–MS. Results show that senescent fibroblasts produced and secreted higher amounts of Gln compared to control fibroblasts, and that labeled Gln is efficiently uploaded by PC3 following conditioning with senescent CM (Figure 5).

### 3.4. Senescent Stroma-Derived Gln Drives the Invasive Abilities of Cancers Cells via a NRF2/ETS1 Axis

**Figure 6:**
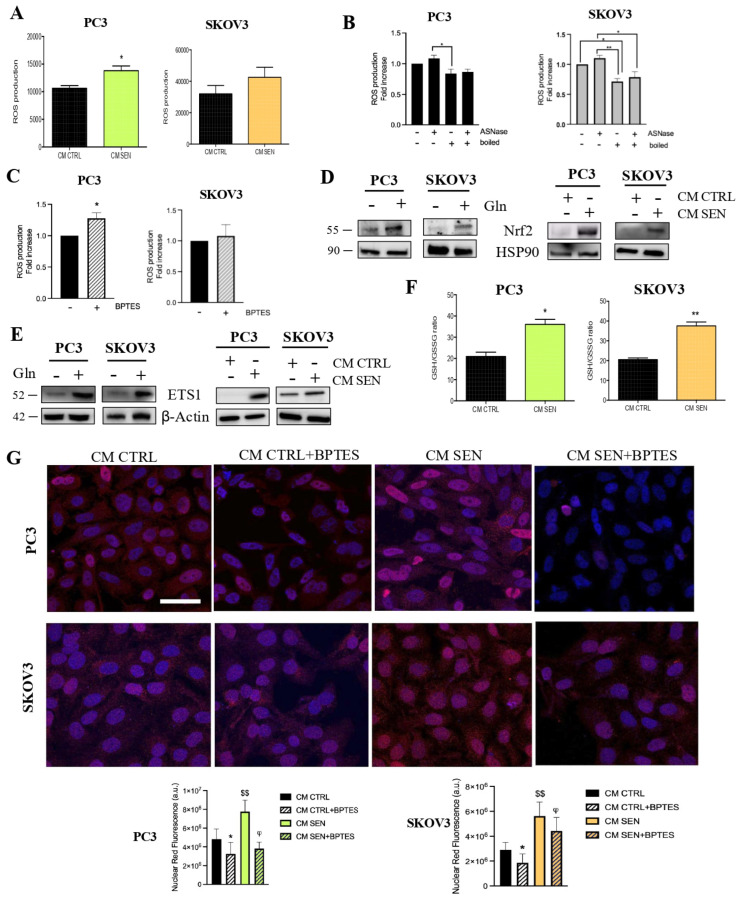
Gln-dependent NRF2/ETS1 pathway drives the invasive abilities of cancer cells. (**A**) Total ROS production was measured with a DCFDA probe in PC3 and SKOV3 cells after 72 h incubation with CM CTRL and CM SEN. (**B**) Total ROS production measured with DCFDA probe in PC3 and SKOV3 cells after incubation with boiled and non-boiled CM CTRL for 72 h in the presence or absence of ASNase 1 U/mL during the last 48 h. (**C**) Total ROS measured as described above. Cells were conditioned for 72 h, BPTES 1 μM was added during the final 16 h of incubation. (**D**) NRF2 protein level in PC3 and SKOV3 cells incubated either with DMEM in presence or absence of 2 mM Gln with CM CTRL and CM SEN. HSP90 immunoblot was performed to ensure equal loading. (**E**) ETS1 protein level in PC3 and SKOV3 cells incubated either with DMEM in presence or absence of 2 mM Gln or with CM CTRL or CM SEN. Actin was used as loading control. (**F**) GSH/GSSG ratio in prostate and ovarian cancer cells incubated for 72 h with CM CTRL or CM SEN. Data are normalized to protein content. (**G**) PC3 and SKOV3 cells were incubated for 72 h with CM CTRL or CM SEN; BPTES 1 μM was added during the final 16 h of incubation. Representative confocal microscopy images show ETS1 nuclear translocation. Bar graphs show the nuclear fluorescence intensity of ETS1 signal. Fluorescence intensity was quantified with ImageJ software (red: ETS1, blue: DAPI; scale bar 44 μm). Data are mean ± SEM of three independent experiments. Statistical significance was assessed by Student *t*-test (**A**,**C**,**F**) or one-way Anova followed by Tukey’s multiple comparisons (**B**,**G**). * *p* < 0.05; ** *p* < 0.01; ^φ^ *p* < 0.05; ^$$^ *p* < 0.01. In panel (**G**), * indicates CM CTRL + BPTES vs. CM CTRL, ^$^ indicates CM SEN vs. CM CTRL, ^φ^ indicates CM SEN + BPTES vs. CM SEN.

It is well known that Gln plays a key role in the antioxidant defence, serving as a critical precursor for the synthesis of GSH, the major cellular antioxidant defence, thereby counteracting the excessive increase of ROS [19,20]. Unexpectedly, we found that treatment with non-boiled CM collected from senescent fibroblasts induced increase ROS levels in prostate and ovarian cancer cell lines (Figure 6A). Conversely, boiled CM inhibited ROS production, suggesting that the cytokine component is the main determinant of the pro-oxidant effect of CM (Figure 6B). Notably, ASNase-mediated depletion of Gln from the non-boiled CM restores ROS production (Figure 6B), supporting the idea that the higher Gln availability in senescent CM can help mitigate the ROS increase caused by the cytokine component. Similar results are observed upon inhibition of intracellular Gln utilization; as shown in Figure 6C, treatment with BPTES leads to an increase in ROS levels following conditioning. In line with these data, Gln addition to the medium is associated to an antioxidant response dependent on the expression of the NF-E2-related factor 2 (NRF2) (Figure 6D), a transcription factor involved in the regulation of several antioxidant regulating genes, including those involved in the GSH synthesis [20]. Similarly, increased expression of NRF2 was observed in PC3 and SKOV3 cells following incubation with CM from senescent cells (Figure 6D and Supplementary Figure S1) and, concordantly, a strong increase of the GSH/GSSG ratio in cancer cells was detected (Figure 6E). Notably, it is reported that the antioxidant response element (ARE) may recruit NRF2 to promoters of target genes including the *ETS1* promoter [21]. ETS1 is a transcription factor whose nuclear translocation is dependent on Gln availability [22] and able to promote transcription of proteins involved in cancer cell migration and invasion, such as matrix metalloproteinases (MMPs) and vimentin [22,23,24]. We found that both Gln supplementation or treatment with CM from senescent fibroblasts are associated with increased ETS1 protein levels (Figure 6F and Supplementary Figure S1) and nuclear localization, while pretreatment of cancer cells with BPTES prevented its nuclear translocation (Figure 6G). Our results suggest a possible link between stromal-derived Gln, NRF2 expression and ETS1 regulation. Future investigations will help to better define the involvement of the NRF2/ETS axis in the observed increase in invasive behavior.

**Figure 7:**
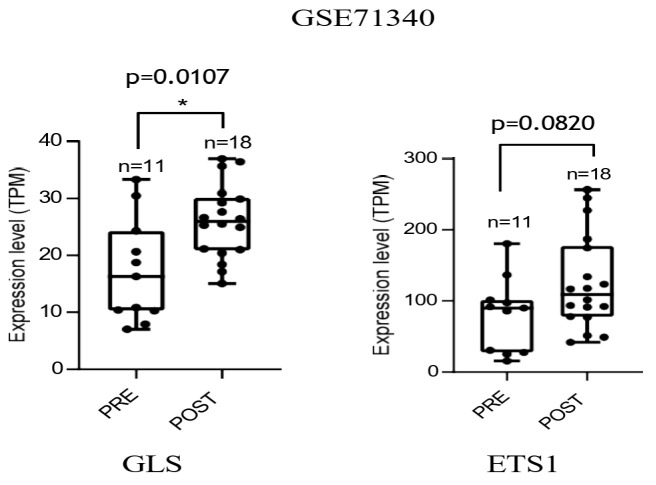
Platinum-based chemotherapy promotes increased expression of *GLS* and *ETS1* in ovarian cancer patients. Gene expression levels of *GLS* and *ETS1* in ovarian cancer tissues pre and post platinum-based therapy from the public RNAseq dataset (GSE71340). Data are presented as median and statistical significance was assessed using a paired, two-tailed Wilcoxon signed-rank test. * *p* < 0.05.

Finally, we evaluated the RNA expression of key enzymes emerged in this study using independent gene expression datasets from the Gene Expression Omnibus (GEO) database. We selected dataset GSE71340, which includes tissue samples of ovarian cancer patients collected before and after platinum-based neoadjuvant chemotherapy (no dataset available for prostate cancer tissues). Public database analyses indicate a significant increase in *GLS* mRNA expression following treatment, along with and upward trend of *ETS1* (Figure 7). Collectively, these data are consistent with a potential association between chemotherapy-associated induction of Gln metabolism and ETS1 activation in patient datasets, which may reflect a contribution of senescence-associated stromal-driven to tumor aggressiveness.

## 4. Discussion

Senescent stroma is increasingly recognized as a key player in modulating cancer progression across several tumor types [25]. Although anticancer therapies can initially restrain tumor growth, relapse is unfortunately common, and recurrent tumors often exhibit a more aggressive phenotype characterized by increased invasiveness, therapy resistance, and metastatic potential. Mounting evidence suggests that TIS-fibroblasts significantly contribute to this unfavorable evolution, as they persist in the TME and, through the acquisition of SASP, create a permissive niche that fosters tumor cell survival and disease progression. While the SASP was originally defined as a complex mixture of secreted proteins, recent evidence indicates that it also includes non-protein factors, such as metabolites and extracellular vesicles, which collectively broaden its impact on the TME.

Here, using metabolic profiling, we identified Gln secreted by TIS fibroblasts as a central non-protein component of the SASP able to enhance invasive and stem-like features in prostate and ovarian cancer cells. To our knowledge, this is the first report showing that TIS fibroblasts are a significant source of extracellular Gln in the TME, highlighting a previously unexplored metabolic mechanism contributing to cancer aggressiveness. Gln is a well-established metabolic driver of tumor progression, with many cancer types described as “Gln-addicted” due to their reliance on this amino acid for energy production, redox balance, and biosynthetic processes [17,26,27]. In addition, Gln metabolism can support resistance to apoptosis [28] and contribute to generate an immunosuppressive tumor microenvironment [11]. Indeed, while previous studies have largely focused on tumor-intrinsic Gln metabolism [15,23,29] and stroma-derived Gln in supporting tumor progression [16,30,31], no data are available regarding the contribution of TIS fibroblasts to Gln availability within the TME. In this context, Gln may represent a paracrine metabolic determinant originating from a therapy-altered microenvironment. Previous data from our lab showed that CM from senescent stroma drives tumor aggressiveness through cytokine-mediated signaling, [7]; here, we extend these findings by uncovering a previously underexplored metabolic dimension of the SASP.

Indeed, our results show that Gln emerged as a metabolite consistently produced across both prostate and ovarian senescent stromal models, pointing to its potential as a shared and functionally relevant metabolic mediator of pro-tumoral roles of TIS fibroblasts. Cancer cell dependency from senescent stroma-derived Gln was confirmed by GS silencing in fibroblasts, Gln depletion in CM or pharmacological inhibition of GLS1 in cancer cells, all of which are associated with reduce invasive capacity without affecting viability. Mechanistically, we showed that senescent CM promotes the expression of the Gln transporter SLC1A5, enhances Gln uptake and its intracellular availability, thereby triggering metabolic reprogramming and inducing EMT and stem-like traits—effects. Moreover, Gln may act as a precursor of GSH: indeed, we observed an increased level of the GSH/GSSG ratio following treatment of cancer cells with senescent CM, suggesting that stromal-derived Gln contributes to maintaining redox balance. The observed changes in NRF2 and ETS1 levels [21,32] provides correlative evidence suggesting a potential involvement of this pathway in promoting invasive traits.

Senescent CM elevates ROS levels in cancer cells, primarily through the cytokine/protein components. The concomitant presence of Gln in the CM appears to be associated with a modulation of this oxidative stress, accompanied by an antioxidant response via NRF2 activation/ETS1 expression and its nuclear translocation in a Gln-dependent manner [22]. Given that ETS1 has previously been reported to be involved in EMT, migration, and invasion [33,34], our data suggest a possible association between Gln availability and a redox-sensitive transcriptional programs that may be linked to cancer cell invasion. In keeping, in our model BPTES treatment prevents ETS1 nuclear translocation, suggesting a possible association between senescent stroma-derived Gln and cancer cell invasion, potentially involving the NRF2/ETS1 axis.

Of note, a limitation of the present study is that the selected cancer cell lines are highly aggressive. For this reason, the reported effects of senescent stroma-derived Gln on invasion may not entirely reflect the behavior of earlier-stage or more heterogeneous tumors. Extending these experiments to less aggressive cell lines, patient-derived primary cancer cells, or in vivo models will be critical to confirm the generalizability of our findings. However, the clinical relevance of our findings is in part sustained by results obtained from publicly available datasets of tissues from ovarian cancer patients. This analysis revealed that samples collected post platinum-based neoadjuvant chemotherapy display a trend to increase the expression of *GLS1* and *ETS1* compared to corresponding tissues collected before therapy intervention. Although they provide only partial clinical validation, these data are consistent with our mechanistic observations. Further validation in independent clinical samples will be required to confirm these findings and strengthen their translational relevance.

Given the emerging interest in targeting Gln metabolism to halt cancer progression (particularly through GLS inhibitors currently in clinical trials) [35], our findings suggest that modulating stromal-derived Gln availability might influence tumor cell aggressiveness. These observations warrant further investigation in preclinical co-culture or in vivo models to assess whether targeting stromal Gln could mitigate the pro-tumorigenic effects of TIS.

## 5. Conclusions

In summary, our study highlights the contribution of the metabolic component of the senescent stroma to the acquisition of a more invasive phenotype, with a particular involvement of Gln sustaining the aggressive potential of prostate and ovarian cancer cells. These results suggest the potential relevance of targeting Gln metabolism, in combination with standard chemotherapeutics, to limit tumor relapse and progression.

## Data Availability

The original contributions presented in this study are included in the article/Supplementary Materials. Further inquiries can be directed to the corresponding authors.

## Supplementary Materials

The following supporting information can be downloaded at: https://www.mdpi.com/article/10.3390/cells15090770/s1. Figure S1: Protein level quantification. Bargraphs represent quantification of target protein level compared to relative housekeeping and show the mean of ± SEM of three independent experiments. *t*-test * < 0.05. Figure S2: Secreted metabolites in CM from senescent and control HPFs and HOFs analysed with GC-MS. Peak areas were normalized to cell number. Red circles indicate metabolite levels significantly increased both in senescent CM from HPFs and HOFs. Data are represented as mean±SEM; unpaired multiple t-test was performed with GraphPad. * *p* ≤ 0.05. Figure S3: (A) Gln and ammonium concentration in DMEM medium before and after tratment with 1U/mL ASNase for 48h. (B) Cell viability of PC3 and SKOV3 cells incubated with or without 2mM Gln for 72h. (C) Cell viability of PC3 and SKOV3 cells incubated in starvation medium for 48h in presence or absence of 1 U/mL ASNase D) Cell viability of PC3 and SKOV3 cells incubated in starvation medium for 48h in presence or absence of 1 μM BPTES. Cell viability was measured with LIVE/DEAD staining. Data are represented as mean ± SEM of three independent experiments. Unpaired t-test was performed with GraphPad. **** *p* ≤ 0.001.

## Abbreviations

The following abbreviations are used in this manuscript:

ASNase	L-asparaginase
CM	Conditioned media
CPT	Cisplatin
DTX	Docetaxel
EMT	Epithelial-to-mesenchymal transition
Gln	Glutamine
GLS1	Glutaminase-1
GS	Gln synthetase
HOFs	Human ovarian fibroblasts
HPFs	Human prostate fibroblasts
ROS	Reactive oxygen species
SASP	Senescence-associated secretory phenotype
TIS	Therapy-induced senescence
TME	Tumor microenvironment
