# Supplementary material for: Senescent Stroma-Derived Glutamine: A Driver of Aggressiveness in Prostate and Ovarian Cancer Cells
Source: Cells. 2026 Apr 24;15(9):770. doi: 10.3390/cells15090770 (PMC13163099; doi:10.3390/cells15090770)
Supplement: Supplementary file 1 [file cells-15-00770-s001.zip › cells-4206830-supplementary.pdf]

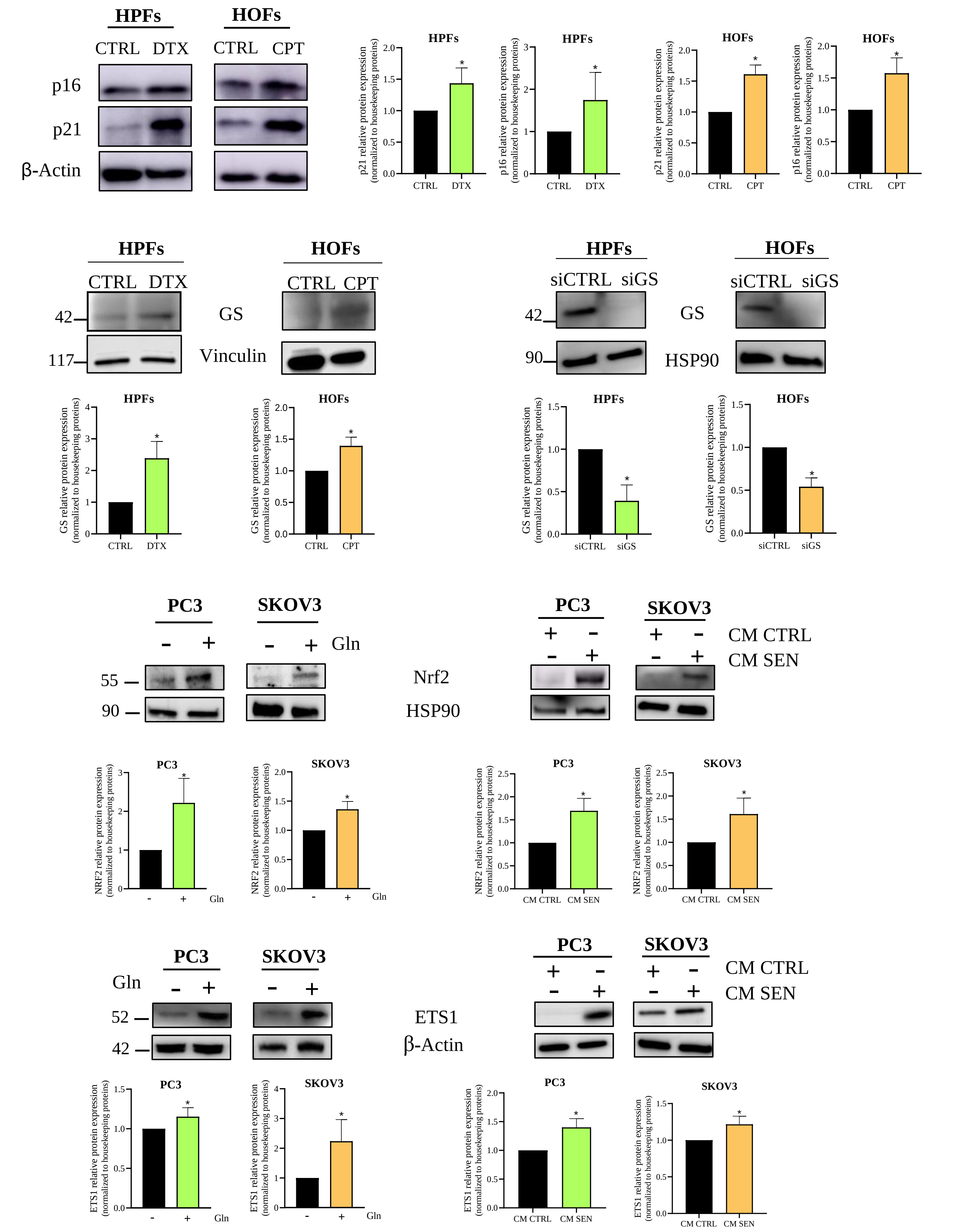

Figure S1: Protein level quantification. Bargraphs represent quantification of target protein level compared to relative housekeeping and show the mean of ± SEM of three independent experiments. t-test *<0,05.

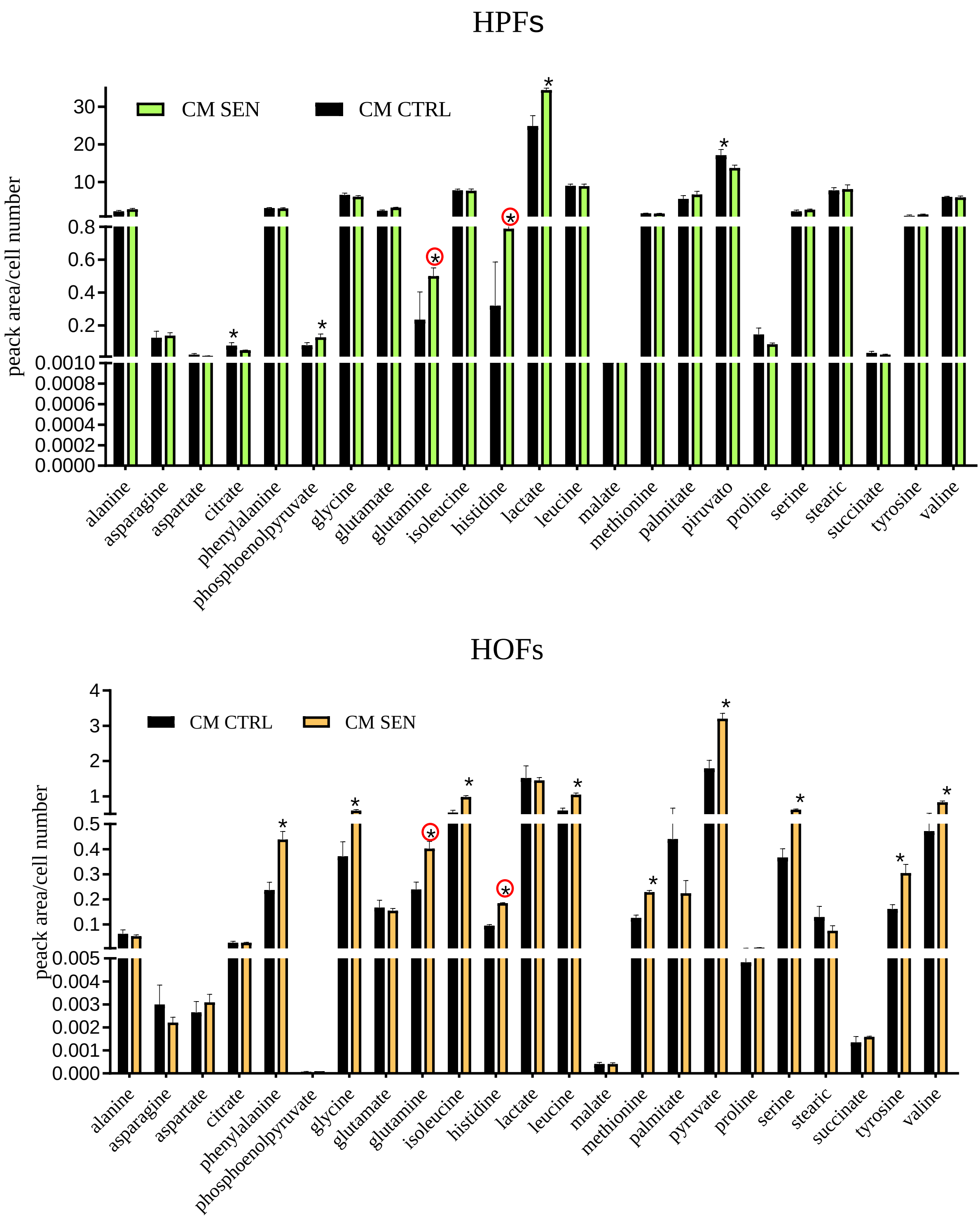

Figure S2: Secreted metabolites in CM from senescent and control HPFs and HOFs analysed with GC-MS. Peak areas were normalized to cell number. Red circles indicate metabolite levels significantly increased both in senescent CM from HPFs and HOFs. Data are represented as mean±SEM; unpaired multiple t-test was performed with GraphPad. *$p \leq 0.05$.

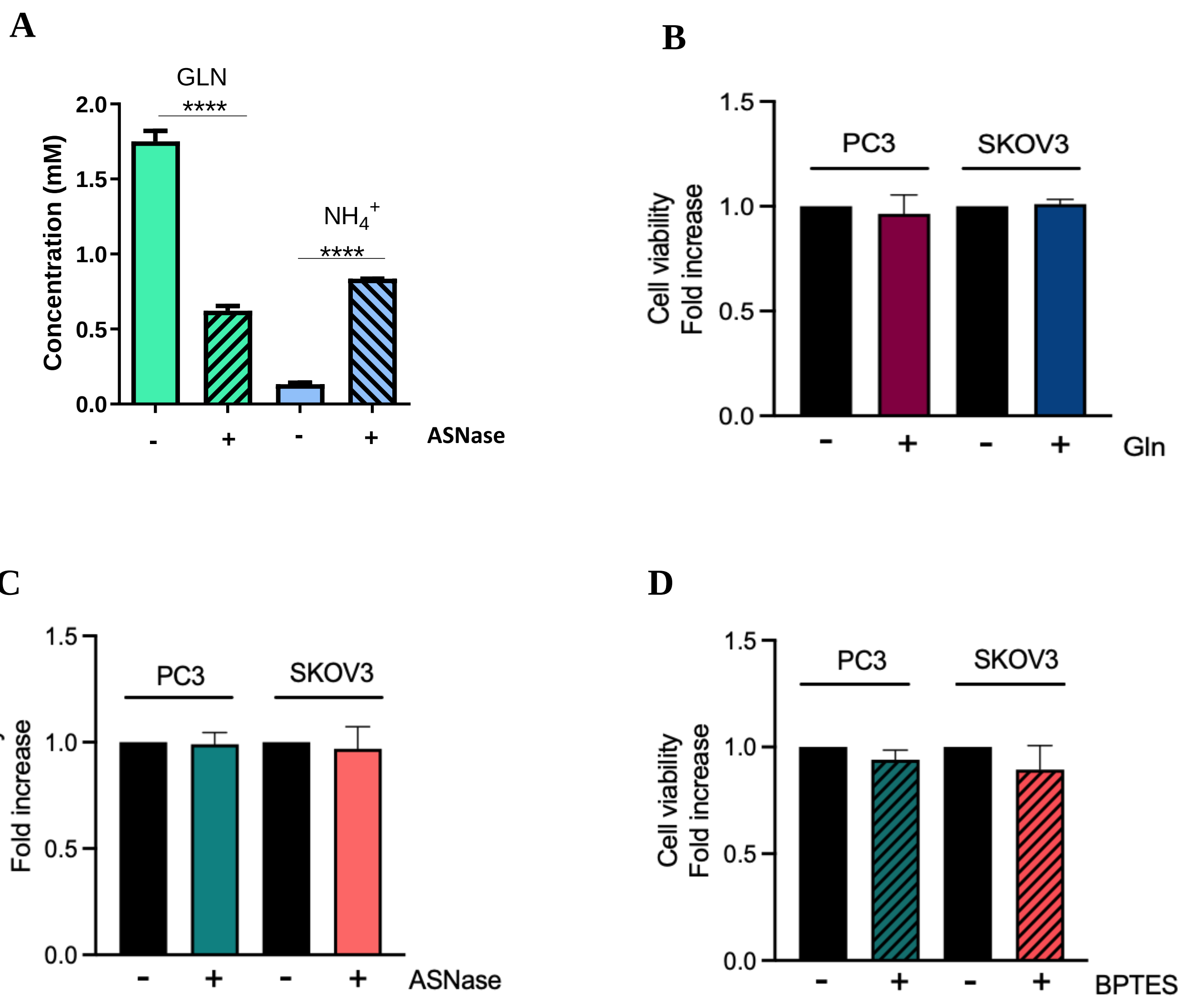

Figure S3: A) Gln and ammonium concentration in DMEM medium before and after tratment with 1U/mL ASNase for 48h. **B)** Cell viability of PC3 and SKOV3 cells incubated with or without 2mM Gln for 72h. **C)** Cell viability of PC3 and SKOV3 cells incubated in starvation medium for 48h in presence or absence of 1 U/mL ASNase **D)** Cell viability of PC3 and SKOV3 cells incubated in starvation medium for 48h in presence or absence of 1μM BPTES. Cell viability was measured with LIVE/DEAD staining. Data are represented as mean ± SEM of three independent experiments. Unpaired t-test was performed with GraphPad. * * * * $p \leq 0.001$.
